# Lipoxygenase in singlet oxygen generation as a response to wounding: *in vivo* imaging in *Arabidopsis thaliana*

**DOI:** 10.1038/s41598-017-09758-1

**Published:** 2017-08-29

**Authors:** Ankush Prasad, Michaela Sedlářová, Ravindra Sonajirao Kale, Pavel Pospíšil

**Affiliations:** 1 Department of Biophysics, Centre of the Region Haná for Biotechnological and Agricultural Research, Faculty of Science, Palacký University, Šlechtitelů 27, 783 71 Olomouc, Czech Republic; 2 Department of Botany, Faculty of Science, Palacký University, Šlechtitelů 27, 783 71 Olomouc, Czech Republic

## Abstract

Wounding, one of the most intensive stresses influencing plants ontogeny and lifespan, can be induced by herbivory as well as by physical factors. Reactive oxygen species play indispensable role both in the local and systemic defense reactions which enable “reprogramming” of metabolic pathways to set new boundaries and physiological equilibrium suitable for survival. In our current study, we provide experimental evidence on the formation of singlet oxygen (^1^O_2_) after wounding of Arabidopsis leaves. It is shown that ^1^O_2_ is formed by triplet-triplet energy transfer from triplet carbonyls to molecular oxygen. Using lipoxygenase inhibitor catechol, it is demonstrated that lipid peroxidation is initiated by lipoxygenase. Suppression of ^1^O_2_ formation in *lox2* mutant which lacks chloroplast lipoxygenase indicates that lipoxygenase localized in chloroplast is predominantly responsible for ^1^O_2_ formation. Interestingly, ^1^O_2_ formation is solely restricted to chloroplasts localized at the wounding site. Data presented in this study might provide novel insight into wound-induced signaling in the local defense reaction.

## Introduction

Various factors are known to affect deleteriously the growth and development in plants^1–3^. Wounding can be related to both biotic and abiotic stresses, as it is caused either by herbivory or plants exposure to environmental mechanical injury^4–6^. Physiological responses of plants to wounding have been categorized based on their timing or spatial distribution. Local responses includes oxidative burst linked with cell wall reorganization or cell death^7, 8^ while systemic response, imply activation of defense related genes^9^, deposition of callose, accumulation of defensive proteins (mostly with enzymatic activity) and lectins^10, 11^. These damage-induced changes are mediated by complex signaling networks, which include receptors, calcium (Ca^2+^) influx, ATP release, kinase cascades, reactive oxygen species (ROS), reactive nitrogen species^12^ and oxylipin signaling pathways^8, 13, 14^. Detailed studies revealed that mechanical injury is tightly linked with variation in electric potential^15, 16^ and chemical signals such as terpenes, methyl salicylate, methyl benzoate, ethylene, and especially jasmonic acid^16^.

Evidence has been provided that wounding leads to the release of polyunsaturated fatty acids from the cell membranes and the accumulation of unsaturated fatty acid at the site of wounding^17^. The activation of phospholipases/other lipases is associated with the release of fatty acids from cell membrane^18^. The released polyunsaturated fatty acid has been known to act as a substrate for lipoxygenase leading to the production of hydroperoxy polyunsaturated fatty acids (lipid hydroperoxide, LOOH)^19^. This enzyme produces precursors for several compounds important for defense reactions, including the plant hormone jasmonic acid^20, 21^. Plant lipoxygenase or linoleate:oxygen 13-oxidoreductase (EC 1.13.11.12) present in different isoforms^22^ is a non-heme iron containing dioxygenase which catalyzes the addition of molecular oxygen to polyunsaturated fatty acid to produce a polyunsaturated fatty acid hydroperoxide. The oxygenation reaction comprises of hydrogen abstraction, radical rearrangement, oxygen insertion and proton addition to polyunsaturated fatty acid^23^. The phospholipase and lipoxygenase activity is categorized as the local response (also referred to as immediate or fast response) which occurs within minutes of wounding in plants which is then followed by the systemic response (also referred to as delayed response) including activation of genes related to phospholipase and lipoxygenase^24^.

**Figure 1:**
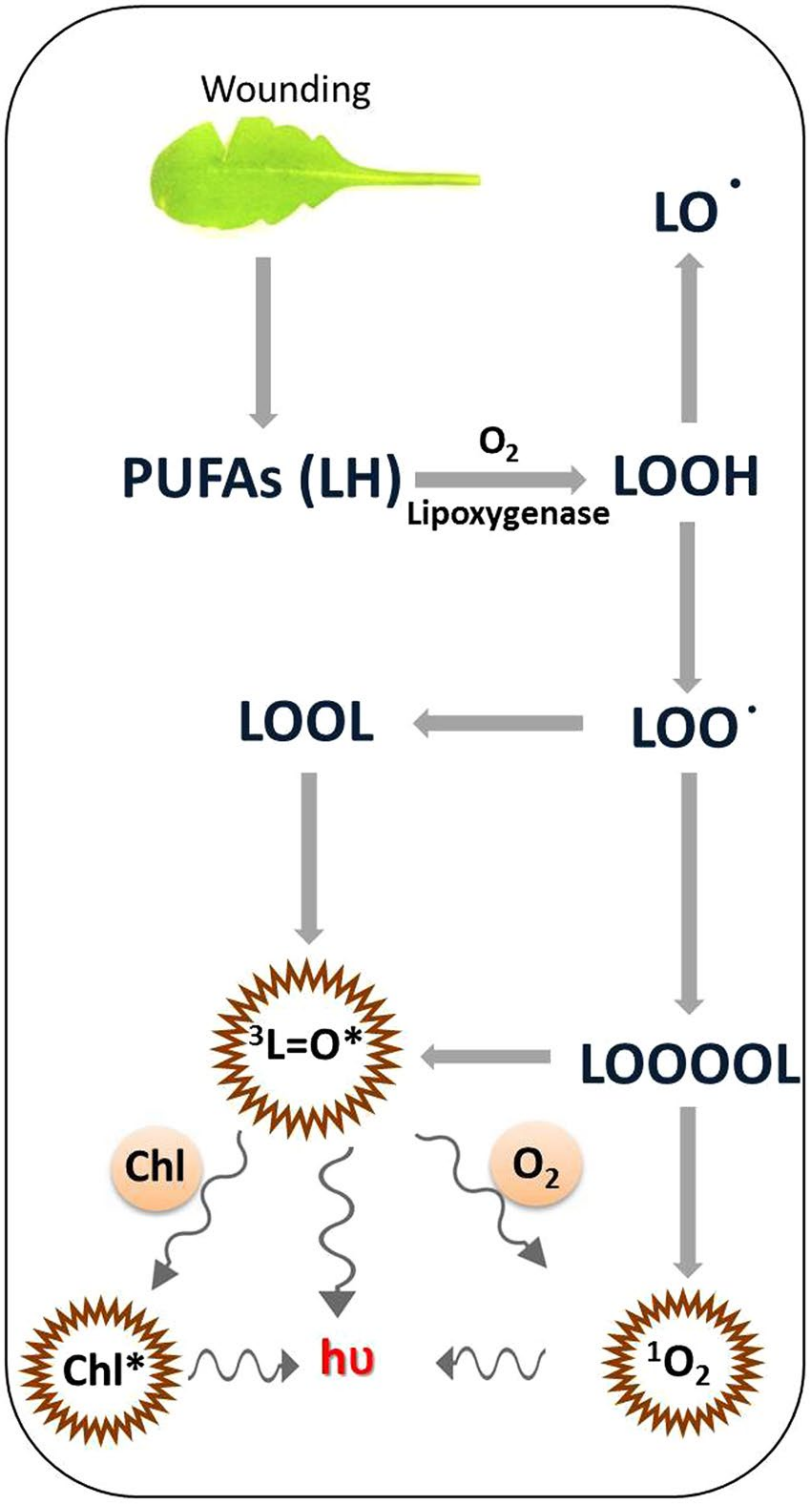
The polyunsaturated fatty acids are oxidized in reaction catalyzed by lipoxygenase subsequently leading to the formation of electronically excited species including triplet carbonyls ﻿﻿(^3^L=O^*^)﻿, singlet chlotrophyll (^1^Chl^*^)﻿ and singlet oxygen (^1^O_2_).

Under reducing conditions, LOOH is reduced by transition metals to lipid alkoxyl radical (LO^•^) which might further abstract hydrogen from another polyunsaturated fatty acid forming another lipid radical (L^•^) and hydroxy polyunsaturated fatty acids (lipid hydroxide, LOH)^25^. Under oxidizing condition, LOOH is oxidized to lipid peroxyl radical (LOO^•^) by oxidized transition metals, ferric heme iron of cytochrome c, peroxynitrite, chloroperoxide, and hypochlorous acid. The cyclization of LOO^•^ is known to form a cyclic endoperoxide (dioxetane), whereas recombination of the two LOO^•^ forms a linear tetroxide^26, 27^. These high energy intermediates decompose to triplet carbonyls (^3^L=O^*^) which might transfer triplet energy either to pigments forming excited pigments or molecular oxygen forming singlet oxygen (^1^O_2_). In addition, tetroxide might decompose directly to^1^O_2_ by Russell mechanism^28–30^ (Fig. 1).

Singlet oxygen imaging with SOSG showed that ^1^O_2_ is formed in the wounded Arabidopsis leaves^31^. The authors proposed that ^1^O_2_ formation is accompanied by recombination of two LOO^•^ via Russell mechanisms formed during enzymatic lipid peroxidation. Nevertheless, no experimental data supporting this proposal have been published yet. In this study, we provide experimental evidence on the role of lipoxygenase in ^1^O_2_ formation after wounding of Arabidopsis plants. Formation of ^3^L=O^*^ monitored by ultra-weak photon emission and ^1^O_2_ detected by the Singlet Oxygen Sensor Green (SOSG) fluorescence assessed by a laser confocal scanning microscopy was studied in *lox2* mutant, which lacks chloroplast lipoxygenase. Our data revealed that in wounded Arabidopsis plants, the lipoxygenase plays a key role in the formation of ^3^L=O^*^ and ^1^O_2_.

## Material and Methods

### Chemical Reagents

All chemicals were purchased of analytical grade from Sigma Aldrich GmbH (Germany) and Molecular Probes Inc. (Eugene, OR, USA).

### Arabidopsis plants

*Arabidopsis thaliana* WT (Columbia-0) and *lox2* mutant lacking chloroplast LOX2 were obtained from Nottingham Arabidopsis Stock Centre^32^, University of Nottingham (Loughborough, United Kingdom). Plants were potted in growing pots with a peat substrate (Klasmann, Potground H) after 4 days of soaking in distilled water. The plants were grown at a photoperiod of 16 h light/8 h dark (photon flux density 100 µmol photons m^−1^ s^−1^) for 6 weeks at a temperature of 25 °C and at 60% relative humidity. The wounding of plant leaves was performed under diffused green light using sharp blade while any external mechanical pressure was avoided at other parts on the surface of plant leaves. Measurement was performed 20 min after wounding.

### Charge coupled device imaging

Highly sensitive CCD camera VersArray 1300B (Princeton instruments, Trenton, NJ, USA) was used for two-dimensional photon imaging. Dark current of the CCD camera was achieved by cooling it down to −110 °C using a liquid-nitrogen cooling system. Spectral sensitivity of the CCD camera was within the range of 350–1000 nm. The quantum efficiency was almost 90% in the visible range of the spectrum. The measurement was done in the image format of 1340 × 1300 pixels and the data correction was done by subtracting the background noise from every measurement. The following CCD camera parameters were used: scan rate, 100 kHz; gain, 2 and accumulation time of 20 min, based on the parameters described in Prasad *et al*.^33^. The CCD camera was situated in a black box located in an experimental dark room with an approximate dimension of 3 m × 1.5 m × 5 m. To avoid any possible interference by external light, the data recording computer was installed in the outer dark room. Prior to the two-dimensional ultra-weak photon emission measurements, the Arabidopsis plant was dark-incubated for approximately 2 h duration to prevent any intervention of delayed luminescence. The data accumulation from Arabidopsis plants and leaves were started 20 min after wounding.

### Confocal laser scanning microscopy

Imaging of ^1^O_2_ was achieved using Singlet oxygen sensor green (SOSG). Wounding of Arabidopsis leaves was exerted by a sharp razor blade used to cut ca 5 × 5 mm pieces from marginal leaf lamina while avoiding other mechanical injury. These cuts were incubated in the presence of 50 µM SOSG for 30 min, washed in 40 mM HEPES buffer (pH 7.5) and forthwith visualized by Fluorview 1000 confocal laser scanning microscope (Olympus Czech Group, Prague, Czech Republic). The excitation was achieved by a 488 nm line of an argon laser and the emission recorded using a 505–525 nm band-pass filter. Negative controls were treated with 5 mM catechol during the staining procedure. Integral distribution of fluorescence signal intensity corresponding to singlet oxygen localization within figures was visualized in FV10-ASW 4.0 Viewer software (Olympus).

## Results

### Triplet carbonyl formation in WT Arabidopsis monitored by ultra-weak photon emission

**Figure 2:**
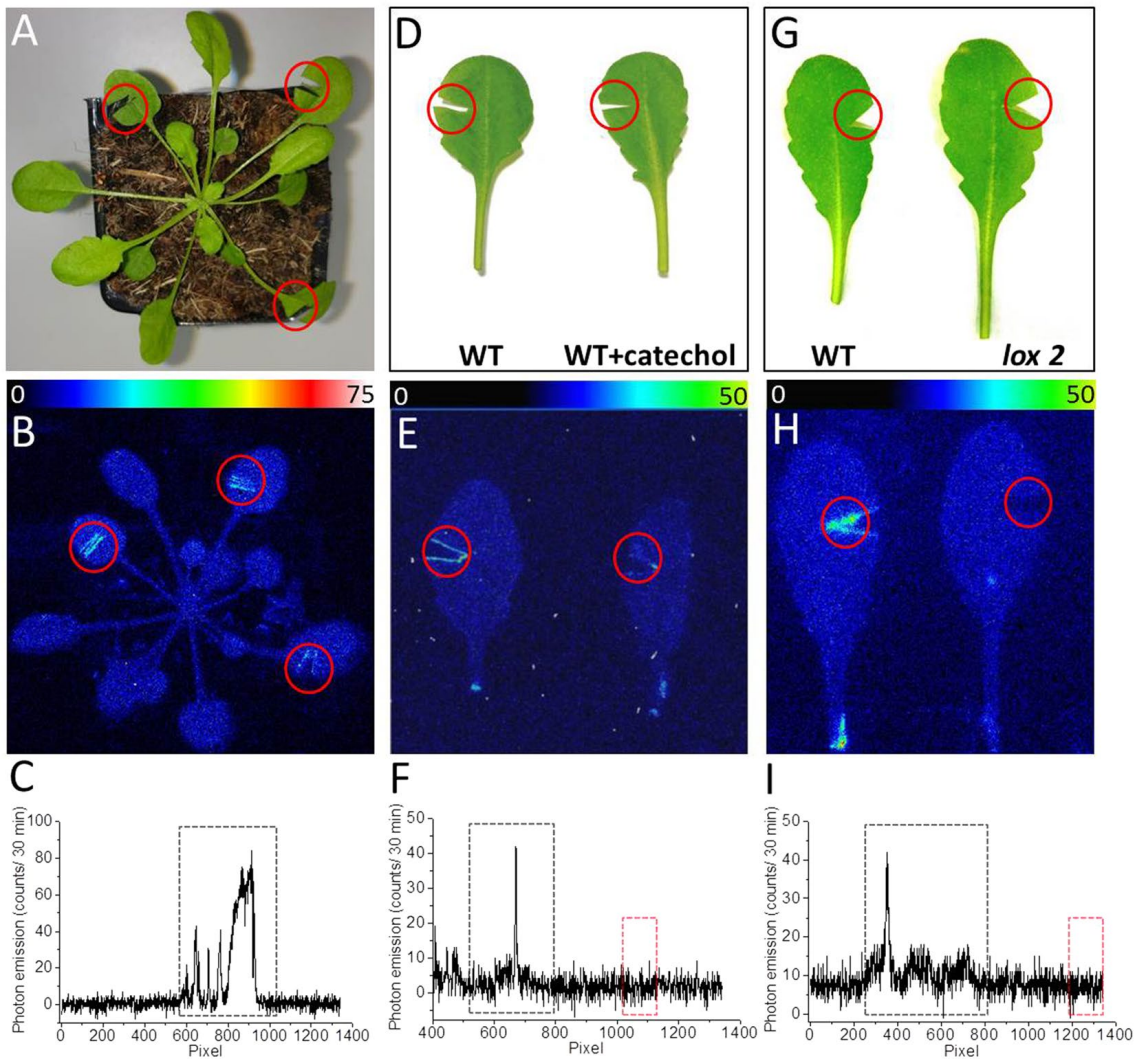
Two-dimensional imaging of the ultra-weak photon emission from the Arabidopsis plant and leaves. The ultra-weak photon emission was measured utilizing highly sensitive CCD camera. The photographs (**A**,**D** and **G**) and the corresponding two-dimensional images of ultra-weak photon emission (**B**,**E** and **H**). In B, two-dimensional images of spontaneous ultra-weak photon emission from non-injured and mechanically injured part of the leaves of Arabidopsis was measured. In E, ultra-weak photon emission imaging was measured in the absence (left leaf) and presence of 5 mM catechol (right leaf) in WT Arabidopsis leaves and in H; ultra-weak photon emission imaging was measured in WT (left leaf) and *lox2* mutants (right leaf) of Arabidopsis leaves. Prior to the measurements, the Arabidopsis plants and leaves were kept in the complete darkness for a period of 2 hrs. The circles in red indicate the mechanically injured part of the leaves. Ultra-weak photon emission imaging was measured after 20 min of wounding with an integration time of 20 min. In C,F and I, the spatial profile of photon emission in a particular strip of the image [Y = 433; Y = 657 and Y = 594, respectively] is presented. Y-axis denotes the number of photon counts accumulated after 20 min, whereas the X-axis denotes pixel of the image. In C, the dotted rectangle represents the position within the Arabidopsis plants at the point of mechanical injury. In F, the dotted rectangle represents the position at the point of mechanical injury (black dotted rectangle) and mechanical injury + catechol (red dotted rectangle) in WT Arabidopsis leaves and in I, the dotted rectangle represents the position within the Arabidopsis leaves at the point of mechanical injury in WT (black dotted rectangle) and *lox2* mutant (red dotted rectangle). The evaluation was performed by transporting the photon intensity at pixel points for the total image frame (1300 × 1340 pixels).

Formation of ^3^L=O^*^ in WT Arabidopsis plants subjected to wounding was monitored by two-dimensional imaging of ultra-weak photon emission. It is well established that singlet chlorophylls (^1^Chl^*^) contribute predominatly to photon emission in plant tissue^27^. As ^1^Chl^*^ is formed solely by excitation energy transfer from ^3^L=O^*^ to chlorophylls, ultra-weak photon emission might serve as an indirect indicator of ^3^L=O^*^ formation. Figure 2 shows a photograph (A) and two-dimensional image of ultra-weak photon emission (B) measured in non-wounded and wounded leaves. Spontaneous ultra-weak photon emission observed from the non-wounded area of Arabidopsis plants is caused by oxidative metabolic processes. The wounding of Arabidopsis plant (Fig. 2A, red circles; Supplementary data 1) resulted in the enhancement of ultra-weak photon emission from the cut edges of the leaves which are caused by wound-induced oxidative processes (Fig. 2B, red circles). The spatial profile of photon emission shows a higher photon emission at the injured part (Fig. 2C). This observation indicates that wounding of Arabidopsis plants results in ^3^L=O^*^ formation restricted solely to the wounded areas of plant. To verify the contribution of the propagative reaction and associated ultra-weak photon emission, we have measured photon emission up to 2 h after wounding (Supplementary data 2). It can be observed that the ultra-weak photon emission as a response to wounding last in the scale of hours. Thus, it can be hypothesized that the ultra-weak photon emission originates at the initial stage due to burst of oxidative reactions after injury and propagative reaction continuing up to few hours.

### Effect of catechol on triplet carbonyl formation in WT Arabidopsis

To study the involvement of lipoxygenase in ^3^L=O^*^ formation, the effect of lipoxygenase inhibitor catechol on ultra-weak photon emission was studied in WT Arabidopsis leaves. It is well established that binding of catechol to the ferric non-heme iron of lipoxygenase leads to inactivation of the enzyme active site^34^. Figure 2 shows a photograph (D) and two-dimensional imaging of ultra-weak photon emission (E) measured in wounded Arabidopsis leaves in the absence (left) and presence (right) of catechol. The topical application of catechol on the wounded areas of the Arabidopsis leaves suppressed significantly ultra-weak photon emission (Fig. 2E and F). The spatial profile of photon emission measured in the presence of catechol (red dotted rectangle) shows that photon emission was decreased to the level of photon emission observed in the non-wounded site (Fig. 2F). As a control, the exogenous application of catechol (n = 3) was also tested in the non-wounded leaves which showed no changes in ultra-weak photon emission (Supplementary data 3). These results reveal that lipoxygenase is involved in ^3^L=O^*^ formation in Arabidopsis leaves exposed to wounding.

### Formation of triplet carbonyls in lox2 mutant

To further clarify the involvement of lipoxygenase in ^3^L=O^*^ formation during wounding of Arabidopsis leaves, two-dimensional imaging of ultra-weak photon emission was measured in WT and *lox2* mutant lacking chloroplast lipoxygenase LOX2 (Fig. 2G–H; Supplementary data 4). Spontaneous ultra-weak photon emission from non-wounded site of *lox2* mutant showed no major difference as compared to WT (Fig. 2H). On the contrary, the ultra-weak photon emission at the site of wounding in *lox2* mutant (Fig. 2H, right leaf) was suppressed compared to WT (Fig. 2H, left leaf). The spatial profile of photon emission in *lox2* mutant (red dotted rectangle) shows the intensity comparable with non-wounded site while the wounded site in WT shows comparatively higher photon emission (black dotted rectangle) (Fig. 2I). These observations indicate that chloroplast lipoxygenase LOX2 plays a key role in ^3^L =O^*^ formation in wounded Arabidopsis leaves.

### Singlet oxygen formation in WT Arabidopsis detected by confocal laser scanning microscopy

**Figure 3:**
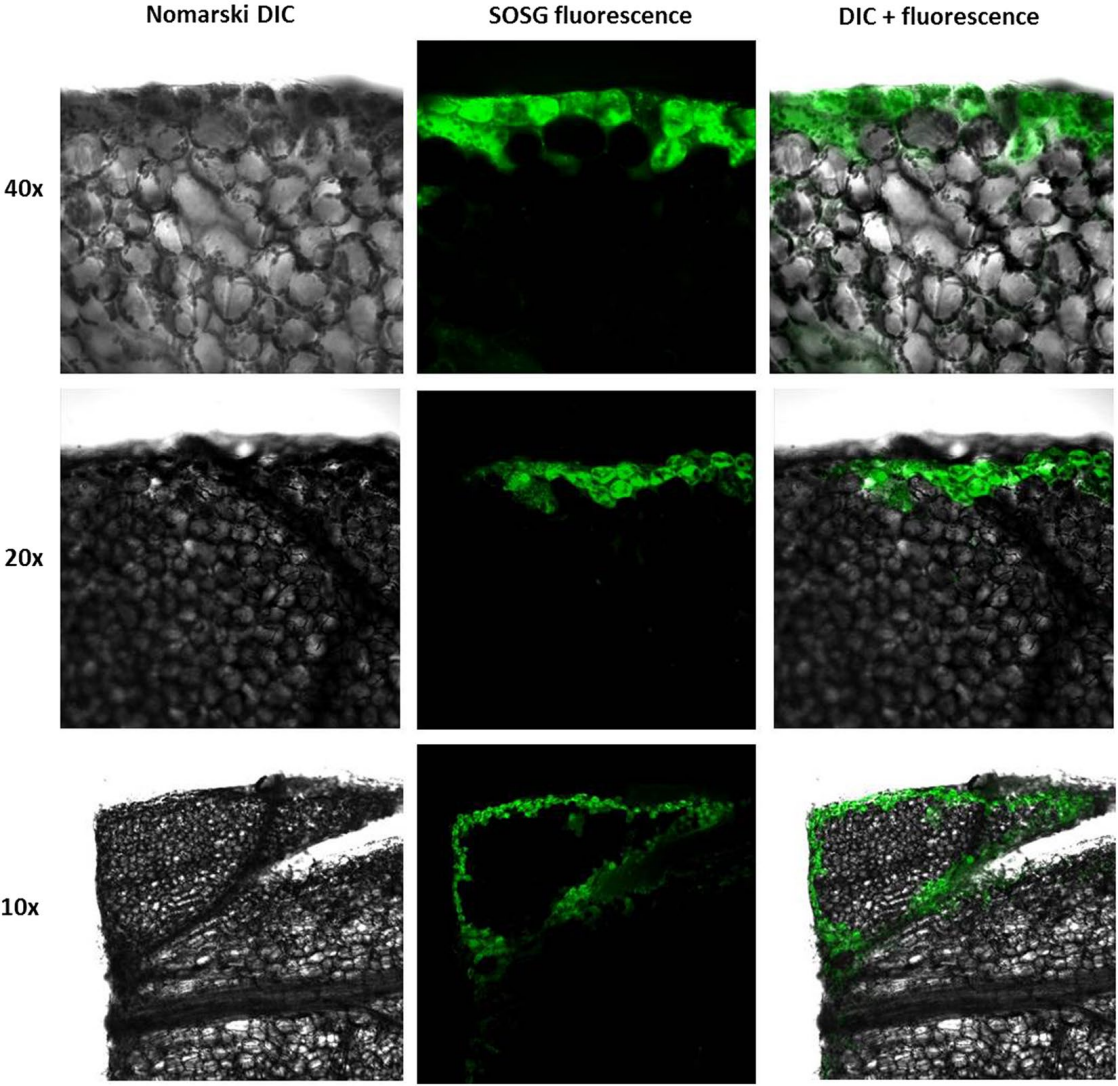
SOSG fluorescence imaging in cells of WT Arabidopsis leaves detected by confocal laser scanning microscope. The left panel represents the DIC; the middle panel represents the SOSG fluorescence and the right panel represents DIC + fluorescence channel following 30 min of incubation in SOSG measured at different magnifications (40x, 20x, 10x) where the margins indicate the wounding site. The fluorescence signal was measured with an excitation (λ_ex_) and emission (λ_em_) wavelengths of 488 nm and 505–525 nm, respectively.

**Figure 4:**
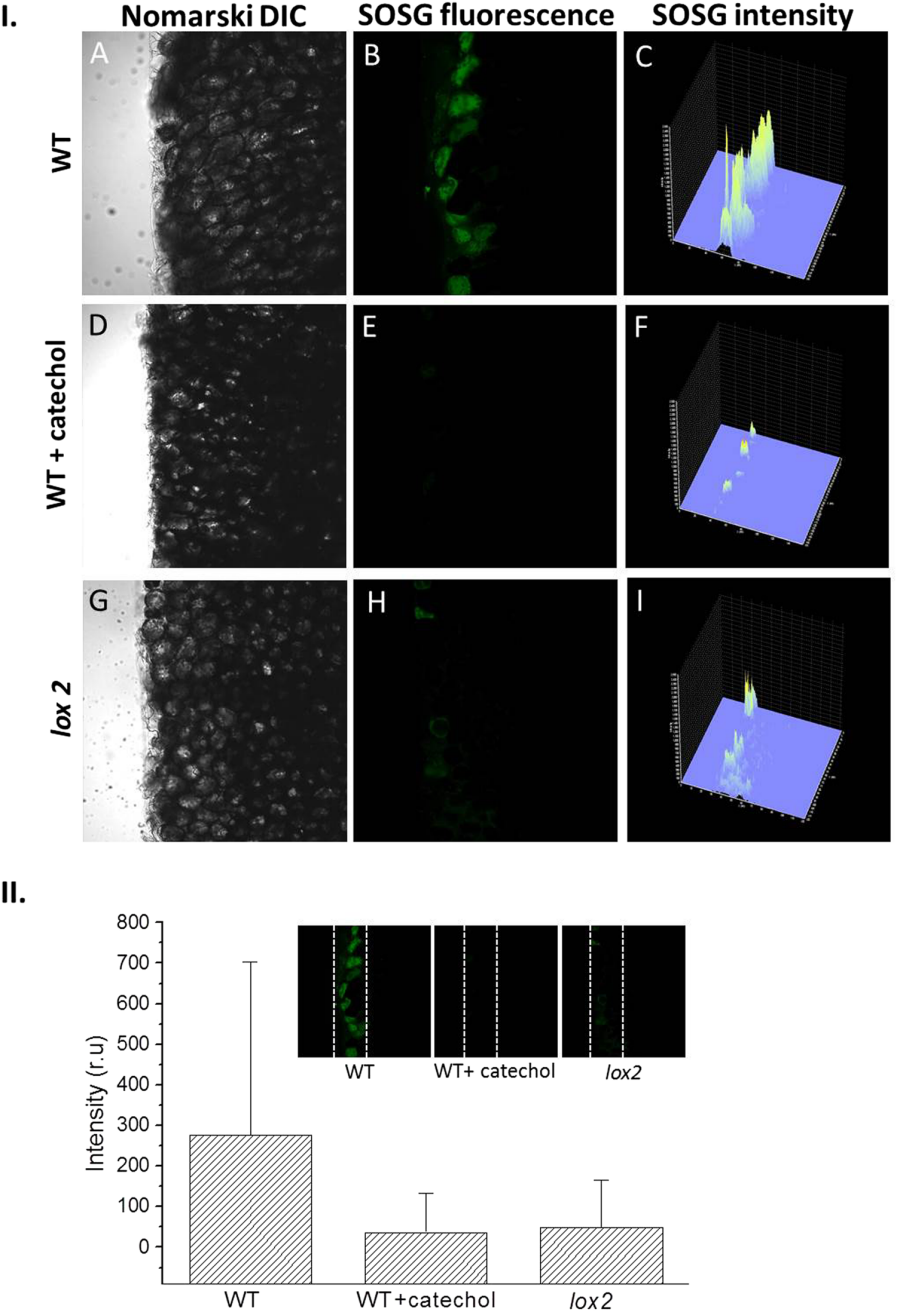
I. SOSG fluorescence imaging in cells of Arabidopsis leaves detected by confocal laser scanning microscope in WT, WT (with catechol) and *lox2* mutant. The left panel represents the Nomarski interference contrast; the middle panel represents the SOSG fluorescence and the right panel represents integral distribution SOSG fluorescence intensity following 30 min of incubation in SOSG. The fluorescence signal was measured with an excitation (λ_ex_) and emission (λ_em_) wavelengths of 488 nm and 505–525 nm, respectively. II. The intensity of the fluorescence signal in SOSG channel of confocal images (800 × 800 pixels, taken under objective magnification 40x) was exported using FV10-ASW 4.0 Viewer software (Olympus). ¼ of the image area was chosen from the cut edge of the leaves (n = 3–5 per each variant) and brightness levels, i.e. values from 0 to 4095, obtained for each of 160 000 px. Following conversion for Microsoft Excel 2010, the data were processed and presented as mean ± standard deviation, completed by maximal signal intensity value.

To visualize the formation of ^1^O_2_ in wounded Arabidopsis leaves, we used SOSG which is highly sensitive and specific fluorescent probe for ^1^O_2_. SOSG fluorescence was detected by confocal laser scanning microscopy. In this method, the formation of SOSG endoperoxide by cycloaddition of ^1^O_2_ to SOSG results in the enhancement of SOSG fluorescence. Figure 3 represents the Differential interference contrast; SOSG fluorescence and DIC + fluorescence channel for comparison of tissue/cell details measured at different magnifications (for objectives 10x, 20x and 40x) where the margins indicate the wounding site which reflects higher SOSG fluorescence signal. SOSG fluorescence was pronouncedly higher in the wounded area compared to none or very low SOSG fluorescence in the intact area of Arabidopsis leaf lamina. Highest SOSG fluorescence signal originated mainly from the first layer of cells on the cutting edge (Fig. 3 and Fig. 4I,A–C). However, the number of cell layers with signal can be influenced by the sharpness of razor blade, i.e. mechanical injury intensity within tissues (Fig. 3, 10X). These observations revealed that wounding in leaves results in ^1^O_2_ formation at the site of injury restricted predominantly to the first, i.e. most impacted, layer of cells and a limited signal from adjoining cells.

### Effect of catechol on singlet oxygen formation in WT Arabidopsis

To confirm the involvement of lipoxygenase in ^1^O_2_ formation caused by wounding, the effect of catechol on SOSG fluorescence was measured in WT Arabidopsis leaves. Figure 4I shows the Nomarski DIC (D), SOSG fluorescence (E) and SOSG intensity (F) measured in wounded Arabidopsis leaves in the presence of catechol. Topical application of catechol on the wounded area of Arabidopsis leaves caused significant suppression of SOSG fluorescence (Fig. 4I,E) as compared to non-catechol treated Arabidopsis leaves (Fig. 4I,B). The distribution of SOSG fluorescence intensity reveals that SOSG fluorescence in wounded Arabidopsis leaf (Fig. 4I,C) was lower as compared to wounded Arabidopsis leaf treated with catechol (Fig. 4I,F). Based on these results, it is concluded that lipoxygenase is involved in the formation of ^1^O_2_ in Arabidopsis leaves under wounding.

### Singlet oxygen formation in lox2 mutant. 

To identify the involvement of lipoxygenase in the formation of ^1^O_2_ during wounding in Arabidopsis leaves, SOSG fluorescence was measured in *lox2* mutant. Negligible SOSG fluorescence was observed in *lox2* mutant at the cut edges of the wounded Arabidopsis leaves (Fig. 4I–H). The distribution of SOSG fluorescence intensity shows that SOSG fluorescence in wounded leaf of *lox2* mutant (Fig. 4I–I) is pronouncedly lower as compared to wounded WT Arabidopsis leaf (Fig. 4I–C). These observations reveal that chloroplast lipoxygenase LOX2 play a key role in ^1^O_2_ formation during wounding in plants. The negligible SOSG fluorescence in few numbers of cells on the cut edge is believed to be contributed by the lipoxygenase located within the cell other than the chloroplasts. Supplementary data 5 and Fig. 4II shows the intensity of SOSG fluorescence channel of confocal images from non-injured edge and the injured edge of WT Arabidopsis leaves, respectively. The results indicate an enhancement in intensity of SOSG fluorescence by 3 times in wounded edge of Arabidopsis leaf as compared to non- wounded areas of the Arabidopsis leaves. The effect to catechol was observed to suppress the SOSG intensity close to the value comparable to non-injured area of the Arabidopsis leaf.

### Effect of desferal and trolox on singlet oxygen formation in WT and lox2 mutant

**Figure 5:**
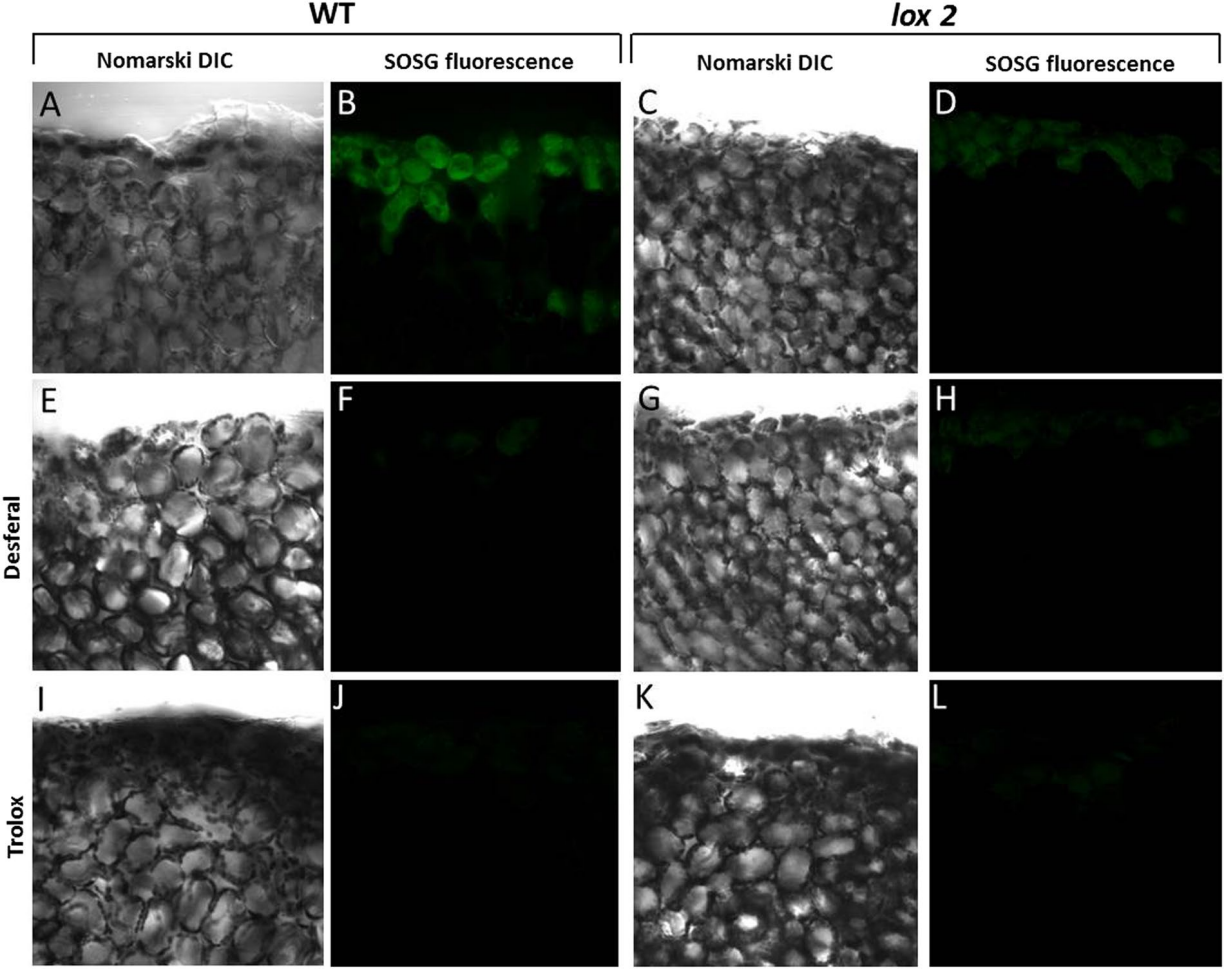
SOSG fluorescence imaging in cells of Arabidopsis leaves detected by confocal laser scanning microscope in WT and *lox2* mutant under the effect of desferal (50 μM) and trolox (4.5 µM). The panels represent the Nomarski interference contrast (NIC) and the SOSG fluorescence following 30 min of incubation in SOSG. All other experimental conditions as in Fig. 4.

The effect of Desferal (deferoxamine mesylate), which is an iron chelator that forms nontoxic ferrioxamine; can attenuate iron-induced oxidative stress and also known to interact with LO^•^ was measured in WT and *lox2* mutant of Arabidopsis leaves. Figure 5 shows the Nomarski DIC [left panel (A, E); right panel (C, G)] and SOSG fluorescence [left panel (B, F); right panel (D, H)] measured in wounded Arabidopsis leaves in WT and *lox2* mutant, respectively. Topical application of desferal on the wounded area of Arabidopsis leaves caused pronounced suppression of SOSG fluorescence in both WT (Fig. 5F) and *lox2* mutant (Fig. 5H) as compared to non-desferal treated Arabidopsis leaves (Fig. 5B and D). As a termination agent for lipid peroxidation, effect of trolox (2-carboxy-2,5,7,8-tetramethyl-6-chromanol) which is a water soluble analogue of vitamin E was measured in WT and *lox2* mutant of Arabidopsis leaves. Topical application of trolox on the wounded area of Arabidopsis leaves caused complete suppression of SOSG fluorescence in both WT (Fig. 5J) and lox2 mutant (Fig. 5L) as compared to non-trolox treated Arabidopsis leaves (Fig. 5B and D).

In addition, ultra-weak photon emission imaging was measured in non-wounded (right leaves of the panel) and wounded (left leaves of the panel) of WT and *lox2* Arabidopsis leaves, respectively in the presence of desferal and trolox showing pronounced suppression in ultra-weak photon emission (Supplementary data 6). Based on these results, it is concluded that inhibition of the propagation and termination step of lipid peroxidation can lead finally to negligible ^1^O_2_ generation.﻿

### Localization of siglet oxygen formation in WT Arabidopsis

**Figure 6:**
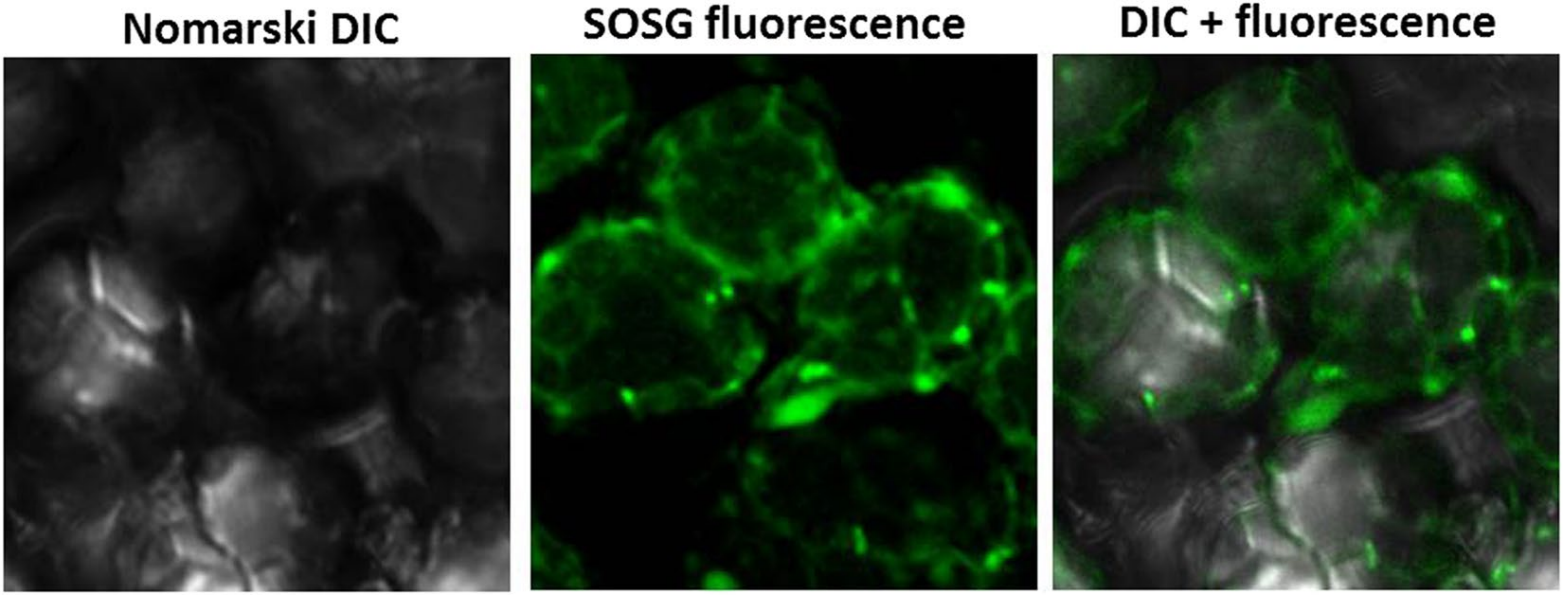
SOSG fluorescence imaging in cells of WT Arabidopsis leaves detected by confocal laser scanning microscope. Nomarski interference contrast (NIC), SOSG fluorescence and NIC + fluorescence channels were measured with an excitation (λ_ex_) and emission (λ_em_) wavelengths of 488 nm and 505–525 nm, respectively. Other experimental conditions as in Fig. 4.

Figure 6 shows the distribution of SOSG fluorescence within the cells at the edges and provides clear evidence indicating the generation of ^1^O_2_ localized predominantly in the chloroplasts. A short videosequence representing Z-stack (2x focusing up and down through the sample) has been presented (Supplementary data 7).

## Discussion

In plants, local response to wounding comprises of oxidative damage of lipids and proteins at the wounding site, whereas systemic response mediated by hormones such as jasmonic acid, ethylene, salicylic acid, and abscisic acid is widespread over the plant tissue and organs^35^. In this study, we provided evidence that ^3^L = O^*^ formed during lipid peroxidation results in ^1^O_2_ formation in the local response to wounding.

### Triplet carbonyl formation

It is known that wounding of plant tissue is accompanied by oxidative damage of lipids and proteins^11^. Two-dimensional imaging of ultra-weak photon emission which is known to be a non-invasive indicator of oxidative stress^36^ was found to be pronouncedly enhanced at the site of wounded plant tissue (Fig. 2; Supplementary data 1). In agreement with our data, Flor-Henry *et al*.^37^ proposed that lipid peroxidation occurs under wounding in detached Arabidopsis leaves using ultra-weak photon emission. More recently, suppression of LOH formation in *lox2* mutant revealed that enzymatic lipid peroxidation is initiated by lipoxygenase^38^. Oxidative burst characterized by ROS production is known to be generated in plant tissues in response to wounding in plants^17, 39^. Recent reports on studies involving *Pisum sativum* and other plant models have claimed activation of NADPH oxidase in response to wounding^17, 39–41^. Evidences have been provided on direct detection of superoxide anion radical (O_2_^•−^) and hydrogen peroxide (H_2_O_2_) measured during wounding in Arabidopsis leaves as monitored by NBT and DAB staining^42^. Due to the fact that light enhanced NBT and DAB signals, the authors proposed that O_2_^•−^ and H_2_O_2_ formation is related to electron transport. In addition, the treatment of Arabidopsis leaves with calcium blockers and calcium chelators after wounding of leaves abolished ROS signal indicating the involvement of calcium in the pathways that couples perception of wounding with the generation of ROS^43^. It has also been reported that LOX2 can be activated by calcium ion; however, its direct interaction is not sufficiently understood^44–47^.

### Singlet oxygen formation

*In vivo* imaging of ^1^O_2_ using SOSG fluorescence measured by confocal laser scanning microscopy revealed that wounding of Arabidopsis leaves caused ^1^O_2_ formation. The observation that lipoxygenase inhibitor catechol completely suppressed ^1^O_2_ formation indicates that lipid peroxidation is initiated by lipoxygenase. Suppression of ^1^O_2_ formation in *lox2* mutant reveals that lipoxygenase localized in chloroplast is predominantly responsible for ^1^O_2_ formation. The observation that ^1^O_2_ formation is localized solely at the site of the wounded plant tissue indicates that ^1^O_2_ unlikely diffuse to surrounding plant tissue. Under dark conditions, the chloroplasts are known to be situated near the periphery attached to the cell membrane of the cells. In the mechanically injured Arabidopsis leaves, the SOSG fluorescence was observed in the periphery close to the cell membrane indicating the generation of ^1^O_2_ localized predominantly in the chloroplasts (Fig. 6). The SOSG fluorescence was observed in layers adjoining the site of mechanical injury indicating that the oxidative radical reaction occurs predominantly close to the site of mechanical injury and that the chain reaction is limited to a close proximity. The termination of chain reaction is likely to occur due to limitation of presence of initiators of the oxidative radical reaction which cannot diffuse to longer distance due to its shorter half-life period. The less probable reason which cannot be neglected completely can be the limited diffusion of the SOSG probe.

### Physiological relevance

Based on the results obtained and understanding from our current study, the response of wounding and generation of ^1^O_2_ can lead to hypothesis on existence of wound induced signaling pathway mediated by ^1^O_2_. The signal observed is predominantly contributed by the chloroplasts which were found suppressed almost entirely in the *lox2* mutant leads to the conclusion that lipoxygenase plays a major role in wound-induced ^1^O_2_ production which is in agreement with experiments performed in model system^48^. However, a lower signal as observed using confocal microscopy may indicate the diffusion of ^1^O_2_ to the neighboring cells. The direct contribution of ^1^O_2_ or oxidized biomolecules can thus be hypothesized to play a role in cellular signaling and opens a new perspective in the signaling pathway^49^. It is proposed here that ^1^O_2_ formed during wounding in plants can be involved in oxidation of either lipids or proteins which can act as signaling molecule.

## Electronic supplementary material

Supplementary data 1–6

Supplementary data 7
