# Supplementary material for: Lipoxygenase in singlet oxygen generation as a response to wounding: in vivo imaging in Arabidopsis thaliana
Source: Sci Rep. 2017 Aug 29;7:9831. doi: 10.1038/s41598-017-09758-1 (PMC5575249; doi:10.1038/s41598-017-09758-1)
Supplement: Supplementary file 1 — Supplementary data 1–6 [file 41598_2017_9758_MOESM1_ESM.pdf]

Lipoxygenase in singlet oxygen generation as a response to wounding: *in vivo* imaging in *Arabidopsis thaliana*

Ankush Prasad[1], Michaela Sedlářová[2], Ravindra Sonajirao Kale[1], Pavel Pospíšil[1]*

[1]Department of Biophysics, Centre of the Region Haná for Biotechnological and Agricultural Research, Faculty of Science, Palacký University, Šlechtitelů 27, 783 71 Olomouc, Czech Republic
[2]Department of Botany, Faculty of Science, Palacký University, Šlechtitelů 27, 783 71, Olomouc, Czech Republic

Corresponding author*
Pavel Pospíšil
Department of Biophysics,
Centre of the Region Haná for Biotechnological and Agricultural Research
Faculty of Science, Palacký University, Šlechtitelů 27, 783 71 Olomouc, Czech Republic
Tel.: +420 585634174; fax: +420 585225737
E.mail: pavel.pospisil@upol.cz

Supplementary data 1

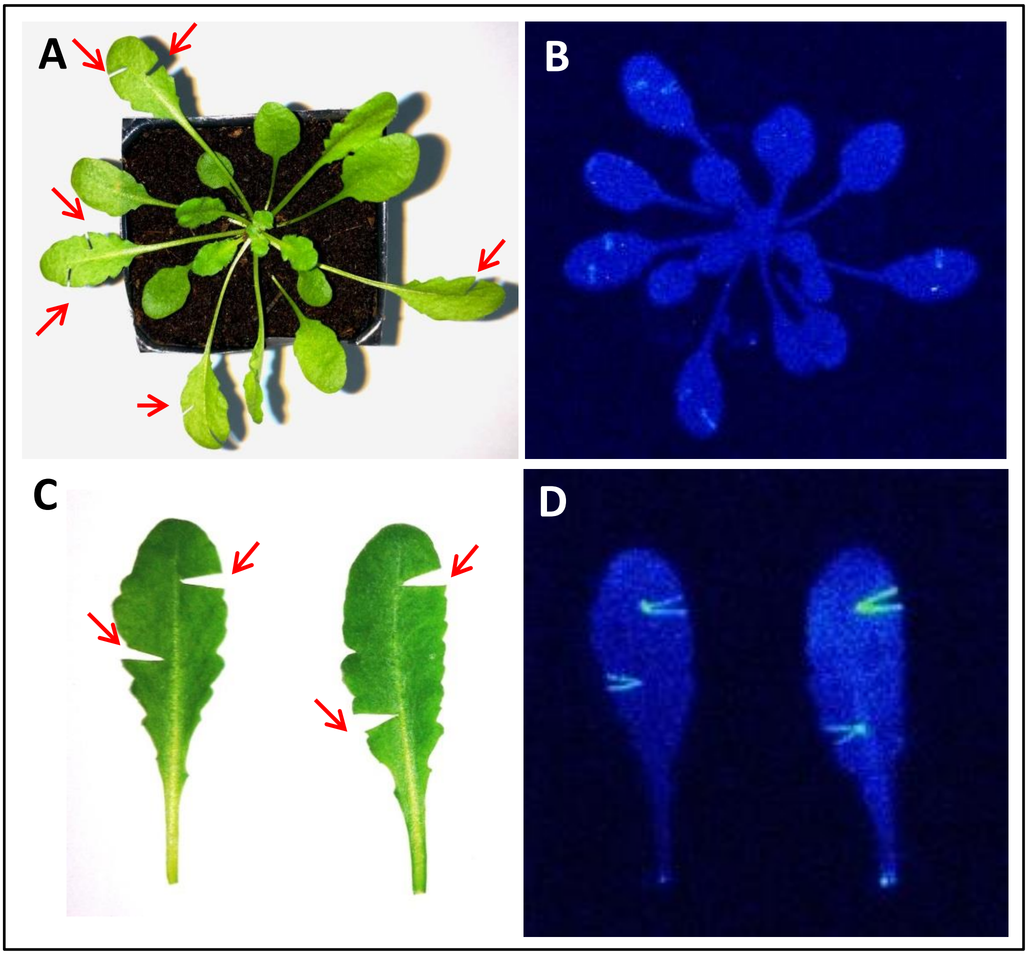

Supplementary data 1: Two-dimensional imaging of the ultra-weak photon emission from *Arabidopsis thaliana*. The ultra-weak photon emission was measured utilizing highly-sensitive CCD camera. The photographs (A, C) and the corresponding two-dimensional images of spontaneous ultra-weak photon emission from Arabidopsis plant and leaves respectively (B, D). Prior to the measurements, the Arabidopsis plants were kept in the complete darkness for a period of 2 hrs. The arrows in red indicate the mechanically injured part of the leaves. Ultra-weak photon emission imaging was measured with an integration time of 20 min.

Supplementary data 2

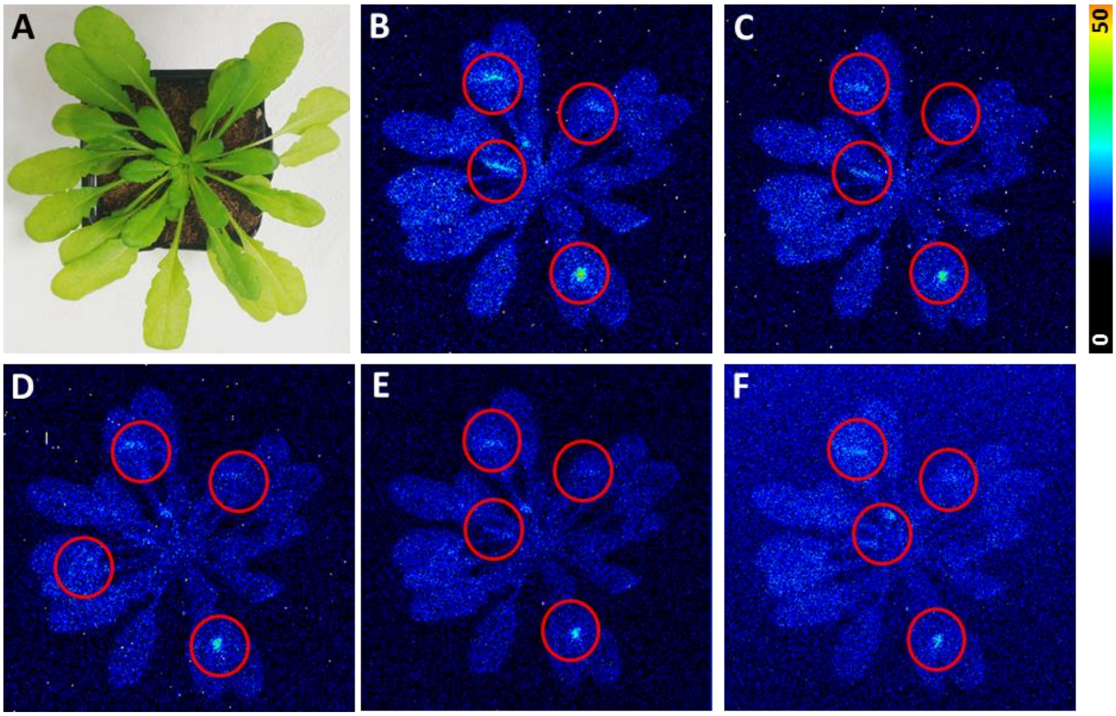

Supplementary data 2: Two-dimensional imaging of the ultra-weak photon emission from the Arabidopsis plant. The ultra-weak photon emission was measured utilizing highly-sensitive CCD camera. The photographs (A) and the corresponding two-dimensional images of ultra-weak photon emission (B-F). In B-F, two-dimensional images of spontaneous ultra-weak photon emission from non-injured and mechanically injured part of the leaves of Arabidopsis were measured at an interval of 20 min each. The circles in red indicate the mechanically injured part of the leaves. Ultra-weak photon emission imaging was measured after 20 min of mechanical wounding with an integration time of 20 min.

Supplementary data 3

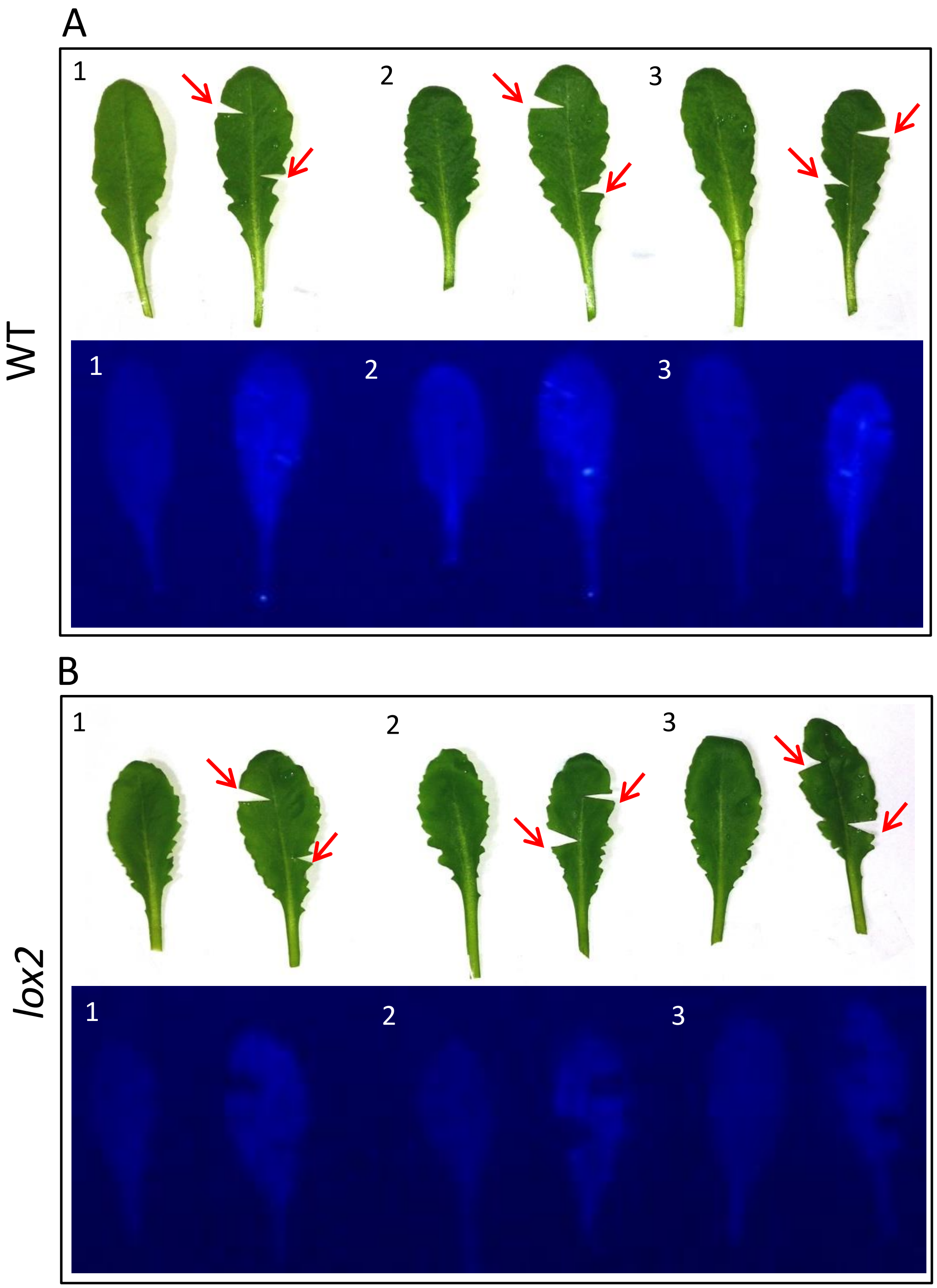

Supplementary data 3: Two-dimensional imaging of the ultra-weak photon emission from the Arabidopsis leaves. The photographs (A, upper panel; B, upper panel) and the corresponding two-dimensional images of spontaneous ultra-weak photon emission (A, lower panel; B, lower panel). Ultra-weak photon emission imaging was measured in non-wounded (left leaves) and wounded (right leaves) of WT and *lox2* Arabidopsis leaves, respectively in the presence of 5 mM catechol (n=3). The arrows in red indicate the mechanically injured part of the leaves. The integration time of 20 min was kept and all other experimental conditions as in supplementary data 1.

Supplementary data 4

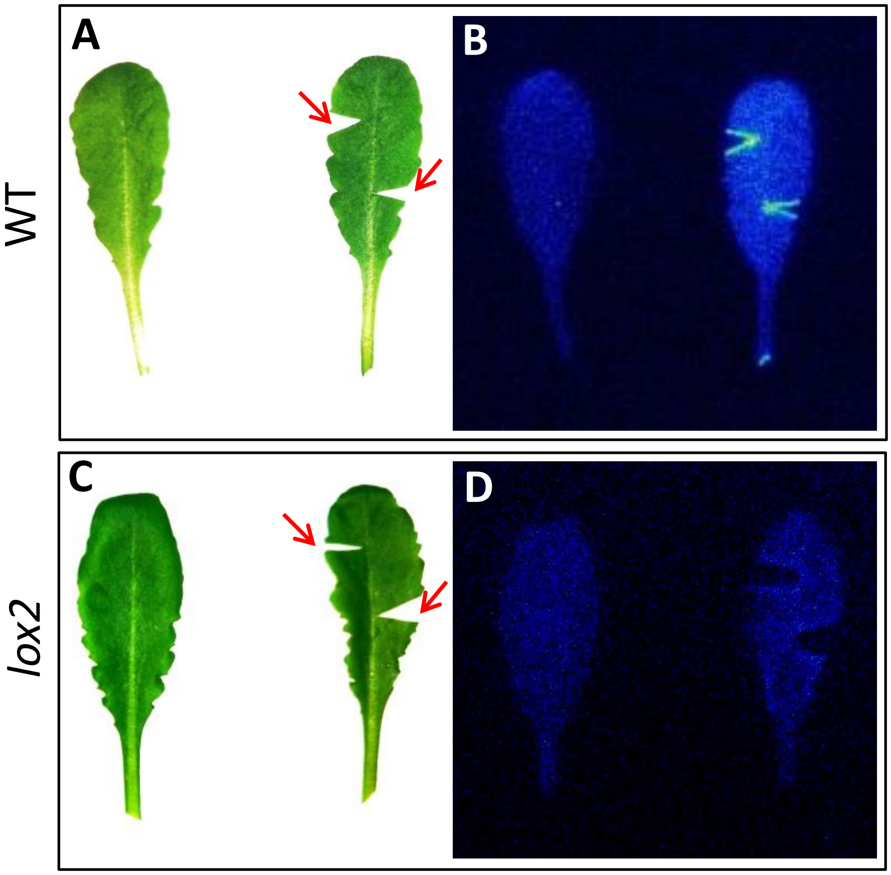

Supplementary data 4: Two-dimensional imaging of the ultra-weak photon emission from the Arabidopsis leaves. The photographs (A, C) and the corresponding two-dimensional images of spontaneous ultra-weak photon emission (B, D). Ultra-weak photon emission imaging was measured in WT (upper panel) and *lox2* mutants (lower panel) of Arabidopsis leaves with an integration time of 20 min. The arrows in red indicate the mechanically injured part of the leaves. All other experimental condition as in supplementary data 1.

Supplementary data 5

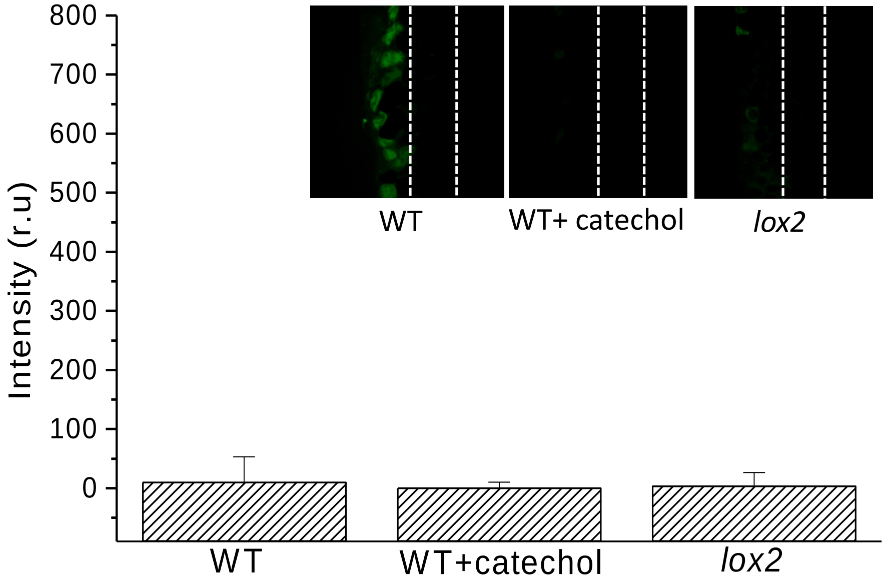

Supplementary data 5: The intensity of the fluorescence signal in SOSG channel of confocal images (800×800 pixels, taken under objective magnification 40x) was exported using FV10-ASW 4.0 Viewer software (Olympus). ¼ of the image area was chosen from non-injured part of the leaves (n = 3-5 per each variant) and brightness levels, i.e. values from 0 to 4095, obtained for each of 160 000 px. Following conversion for Microsoft Excel 2010, the data were processed and presented as mean ± standard deviation, completed by maximal signal intensity value.

Supplementary data 6

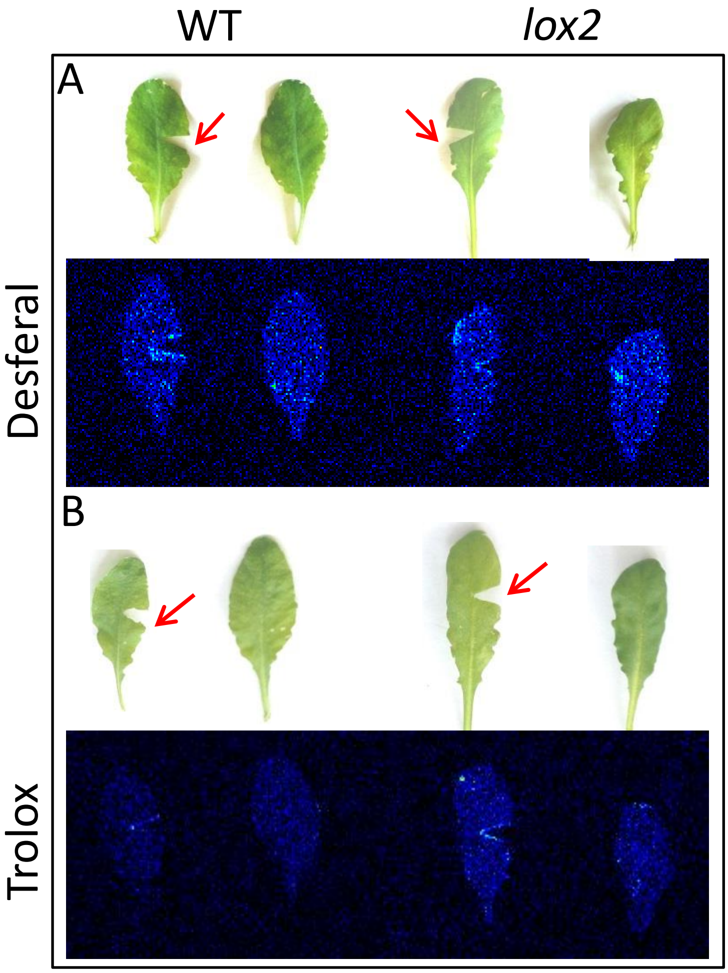

Supplementary data 6: Two-dimensional imaging of the ultra-weak photon emission from the Arabidopsis leaves. The photographs (A, upper panel; B, upper panel) and the corresponding two-dimensional images of spontaneous ultra-weak photon emission (A, lower panel; B, lower panel). Ultra-weak photon emission imaging was measured in non-wounded (right leaves) and wounded (left leaves) of WT and *lox2* Arabidopsis leaves, respectively in the presence of desferal (50µM) and trolox (4.5 µM). The arrows in red indicate the mechanically injured part of the leaves. The integration time of 20 min was kept and all other experimental conditions as in supplementary data 1.
